# Supplementary material for: Patterns of routine primary care for osteoarthritis in the UK: a cross-sectional electronic health records study
Source: BMJ Open. 2017 Dec 29;7(12):e019694. doi: 10.1136/bmjopen-2017-019694 (PMC5778330; doi:10.1136/bmjopen-2017-019694)
Supplement: Author's manuscript [file bmjopen-2017-019694.draft_revisions.pdf]

BMJ Open is committed to open peer review. As part of this commitment we make the peer review history of every article we publish publicly available.

When an article is published we post the peer reviewers' comments and the authors' responses online. We also post the versions of the paper that were used during peer review. These are the versions that the peer review comments apply to.

The versions of the paper that follow are the versions that were submitted during the peer review process. They are not the versions of record or the final published versions. They should not be cited or distributed as the published version of this manuscript.

BMJ Open is an open access journal and the full, final, typeset and author-corrected version of record of the manuscript is available on our site with no access controls, subscription charges or pay-per-view fees (http://bmjopen.bmj.com).

If you have any questions on BMJ Open's open peer review process please email editorial.bmjopen@bmj.com

BMJ Open

Patterns of routine primary care for osteoarthritis: a cross-sectional electronic health records study

Journal:	*BMJ Open*
Manuscript ID	bmjopen-2017-019694
Article Type:	Research
Date Submitted by the Author:	19-Sep-2017
Complete List of Authors:	Jackson, Holly; Keele University, Research Institute for Primary Care and Health Sciences; University of Nottingham, School of Mathematical Sciences<br>Barnett, Lauren; Keele University, Arthritis Research UK Primary Care Centre<br>Jordan, Kelvin; Keele University, Arthritis Research UK Primary Care Centre; Keele Clinical Trials Unit, David Weatherall Building<br>Dziedzic, Krysia; Arthritis Research Campaign National Primary Care Centre<br>Cottrell, Elizabeth ; Keele University, Research Institute for Primary Care & Health Sciences<br>Finney, Andrew; Keele University, Research Institute for Primary Care and Health Sciences<br>Paskins, Zoe; Keele University, Arthritis Research UK Primary Care Centre, Research Institute for Primary Care & Health Sciences<br>Edwards, John; Keele University Faculty of Health, Research Institute for Primary Care & Health Sciences
**Primary Subject Heading**:	General practice / Family practice
Secondary Subject Heading:	Rheumatology
Keywords:	Osteoarthritis, PRIMARY CARE, Quality in health care < HEALTH SERVICES ADMINISTRATION & MANAGEMENT, Latent class analysis

SCHOLARONE™ Manuscripts

Patterns of routine primary care for osteoarthritis: a cross-sectional electronic health records study

H Jackson[1,2], LA Barnett[1], KP Jordan[1,3], KS Dziedzic[1], E Cottrell[1], AG Finney[1], Z Paskins[1], JJ Edwards[1]

H Jackson: holly@gigglane.com

LA Barnett: l.a.barnett@keele.ac.uk

KP Jordan: k.p.jordan@keele.ac.uk

KS Dziedzic: k.s.dziedzic@keele.ac.uk

E Cottrell: e.cottrell@keele.ac.uk

AG Finney: a.finney@keele.ac.uk

Z Paskins: z.paskins@keele.ac.uk

JJ Edwards: j.j.edwards@keele.ac.uk

[1]Keele University Research Institute for Primary Care and Health Sciences; [2]University of Nottingham School of Mathematical Sciences; [3]Keele Clinical Trials Unit, David Weatherall Building, Keele University, Staffordshire, ST5 5BG

Correspondence to: Dr John Edwards, Arthritis Research UK Primary Care Centre, Research Institute for Primary Care & Health Sciences, Keele University, Staffordshire, ST5 5BG; e-mail: j.j.edwards@keele.ac.uk; tel. +44 (0) 1782 733977; fax.: +44 (0) 1782 734719

Word count: 3424

Running title: Patterns of primary care for osteoarthritis

For peer review only

Abstract

Objective

To determine common patterns of recorded primary care for osteoarthritis (OA), and characteristics associated with the quality of recorded care.

Design

An observational study nested within a cluster-randomised controlled trial.

Setting

Eight UK general practices who were part of the Management of OSteoArthritis In ConsultationS (MOSAICS) study.

Participants

Patients recorded as consulting within the eight general practices for clinical OA.

Primary outcomes

Achievement of seven quality indicators of care, recorded through an electronic template or routinely recorded in the electronic healthcare records, were identified for patients aged ≥45 years consulting over a six-month period with clinical OA. Latent class analysis was used to cluster patients based on care received. Clusters were compared on patient and clinician-level characteristics.

Results

1724 patients consulted with clinical OA. Common patterns of recorded quality care were: Cluster 1 (38%, *High*) received most quality indicators of care; Cluster 2 (11%, *Moderate*) had pain and function assessment, and received or were considered for other indicators; Cluster 3 (17%, *Low*) had pain and function assessment, and received or were considered for paracetamol or topical NSAIDs; Cluster 4 (35%, *None*) had no recorded quality indicators. Patients with higher levels of recorded care consulted a clinician who saw more OA patients, consulted multiple times, and had

For peer review only

less morbidity. Those in the *High* cluster were more likely to have recorded diagnosed OA and have knee or hip OA.

Conclusions

Appropriate delivery of core interventions and relatively safe pharmacological options for OA are still not consistently recorded as provided in primary care. Further research to understand clinical recording behaviours and determine potential barriers to quality care alongside effective training for clinicians is needed.

Trial registration number ISRCTN06984617

Keywords

Osteoarthritis, primary care, quality indicators, latent class analysis

Article summary

- This paper describes a novel use of latent class analysis to identify patterns of primary care for osteoarthritis (OA)
- The population studied was large and diverse, increasing generalisability, and based on a broad definition of clinical OA to reduce selection bias
- The analysis used some quality indicators of care newly-implemented in practices through an electronic template, which may have increased the recorded quality of care compared to routine practice
- Some care processes may have occurred but not been recorded

Word count 3424

For peer review only

Introduction

Osteoarthritis (OA) is a common reason for adults aged ≥45 years to consult primary care. Annually, in the UK, 4% of such adults are recorded as consulting in general practice for diagnosed OA, with an additional 8% recorded with joint pain likely to be attributable to OA [1]. Osteoarthritis is a common reason for disability, and was ranked the 11th biggest cause of disability by the 2010 Global Burden of Disease (GBD) [2].

The UK National Institute for Health and Care Excellence (NICE) OA management guidelines recommend core strategies of information provision, physical activity and exercise, and weight management, supplemented with use of relatively safe pharmacological management strategies (for example, topical non-steroidal anti-inflammatory drugs [NSAIDs]), as necessary [3]. Intensification of management should depend on response to these initial approaches. However, there is evidence that patients diagnosed with OA do not receive care that is well aligned to evidence-based recommendations and which may be overly dependent on pharmacological methods [4].

We have previously identified variation between clinicians in recorded quality of individual indicators of OA care [5]. However, patterns of OA care and factors linked with increased probability of adherence to OA quality standards are less well-studied. Using electronic general practice records data, the objectives of this study were to determine patterns of recorded primary care for OA based on quality indicators, and to determine associations between higher-quality recorded care and patient and clinician characteristics.

Methods

This analysis used data from the Management of OSteoArthritis In ConsultationS (MOSAICS) study (Trial registration number ISRCTN06984617), approved by the North West Research Ethics

For peer review only

Committee, Cheshire (reference: 10/H1017/76) [6]. MOSAICS was a mixed-methods study, which investigated the effect of a model consultation for clinical OA. It was set within eight general practices in Cheshire, Shropshire and Staffordshire, UK and is reported in line with STROBE guidelines. The current analysis used anonymised information from the electronic health records (EHR) of these practices for the six-month baseline period before randomisation of practices to intervention or control arms [6]. At the beginning of the baseline period, a computerised template ("e-template", described below) was installed within the EHR and all practices continued with otherwise usual care until the end of the baseline period.

The study population was all patients aged ≥45 years registered with the eight general practices who consulted with clinical OA in the baseline six-month period. UK general practice utilises Read codes to record morbidities; within MOSAICS, clinical OA was defined as either a recorded OA Read code or a peripheral joint pain Read code for the hand, hip, knee, or foot, to reduce the potential for selection bias in clinician coding. Patients were allocated to an index clinician, being the clinician recording the first formally diagnosed (i.e. OA Read-coded) OA consultation in the baseline period or, if none, the first peripheral joint pain coded consultation in the same period.

Outcome measures were the seven indicators of quality of care for OA in general practice recorded in the EHR. These could be entered into the EHR as routinely-recorded data or captured through the e-template. The identification and synthesis of appropriate quality indicators using a systematic review and NICE 2008 guidelines has previously been reported [5,7,8]. The indicators are shown in Table 1.

Achievement of prescribing and referral indicators (recorded prescription of topical NSAIDs or paracetamol, and onward physiotherapy referral) were determined from data in the routinely-

For peer review only

recorded component of the EHR and were determined to have been achieved if they were recorded within 14 days of any clinical OA consultation in the six-month period.

The e-template facilitated recording of achievement of indicators that are known to be poorly captured in routinely-recorded data [5]: (i) assessment of pain and function, (ii) provision or consideration of OA information, exercise advice, and weight loss advice, (iii) consideration of paracetamol or topical NSAID and (iv) consideration of physiotherapy referral. The entry of a code for clinical OA for a patient aged ≥45 years triggered the e-template [5]. The clinicians could complete the e-template at any point throughout the consultation and could choose to complete all, some, or none of the e-template. The e-template has been endorsed by NICE to facilitate enhanced uptake of quality standards [9].

Data from the EHR (derived from both routinely-recorded data and the e-template) were amalgamated within the relevant quality indicator. For example, consideration of paracetamol and topical NSAIDs (entered using e-template) was combined with actual prescription of these agents (routinely-recorded data). Outcomes (Table 1) were dichotomous for pain and function assessments. For all other indicators, the possibilities were for the indicator to be *achieved*, *considered* (without record of having been delivered), or *not considered*. There is evidence that weight recording is more common in people who are overweight compared to those who are not [10]. To minimise the effect of missing data and to preserve the ability of the model to identify people who needed weight loss advice but were not recorded as receiving it, any patient recorded as being of normal weight or who did not have a weight recorded was allocated to *considered* for weight loss advice.

For peer review only

We investigated how patterns of care based on the quality indicators were associated with other OA care processes, recorded in the routine EHR within 14 days of any clinical OA consultation: prescriptions for oral NSAIDs and opioids, and relevant X-rays (hand, hip knee, or foot).

Factors potentially associated with patterns of quality of care that were considered were: patient age, gender, body mass index (BMI), the site of clinical OA, whether patients had multiple or a single consultation for clinical OA within the six-month time period, whether the patient was a new consulter (no clinical OA consultations within the previous 12 months) and total morbidity. Total morbidity was measured by a count of British National Formulary (BNF) subchapters from which prescriptions had been issued in the previous 12 months [11]. A proxy measure of OA workload for the patients' index clinician was determined by dichotomising the number of index clinical OA consultations at the median value (14) across clinicians.

Statistical analysis

Latent class analysis (LCA) was used to cluster patients into groups based on recorded achievement of the seven quality indicators. All patients within a cluster should have similar recorded care for their OA or joint pain, but care should differ between patients belonging to different clusters [12].

Latent class models were fitted, beginning with a one-cluster model where all the patients were assumed to have been given the same pattern of treatment of OA, up to a seven-cluster model. To determine the optimum number of clusters, we considered the Bayesian Information Criterion (BIC, whereby the lowest BIC indicated the best model) with the size of each cluster, and the interpretability of the model. Posterior probabilities (PP) for a patient (the probabilities of that patient belonging to each of the clusters within the model) were identified. The cluster that had the largest PP for a patient was the cluster that patient was assigned to. We used the mean PP for

For peer review only

patients allocated to each cluster to measure cluster separation; a mean PP of more than 0.7 indicated that the patients were clearly assigned to that specific cluster [13].

Using a two-level (patient within index clinician) multinomial multilevel logistic regression, associations between the patient and clinician-level covariates and cluster membership were estimated and reported as relative risk ratios (RRR) with 95% confidence intervals (CI). We also used chi-squared tests to compare between clusters on levels of pain and functional limitation (none, mild, moderate, severe) as recorded in the e-template.

Statistical analysis was performed using R studio version 3.3.0, and MLwiN version 2.35 for Windows.

Results

During the six-month period, 1724 patients consulted with a recorded clinical OA code and triggered the e-template. All were included in the analysis. 1014 (59%) of these were female, mean age was 66.1 years (SD: 11.9) and 582 (34%) patients were recorded with a diagnosis of OA rather than peripheral joint pain.

As previously reported [5], pain (63%) and function (62%) assessment were the most commonly achieved indicators. Recorded provision of OA information (44%), and exercise advice (45%) were achieved in under half of patients, and weight loss advice in less than a third of patients (31%). 609 (35%) patients were prescribed paracetamol or topical NSAIDs. A referral for physiotherapy was made in 7% of patients.

Table 2 shows the goodness-of-fit statistics for the LCA models with one to seven clusters. The four-cluster model gave the lowest BIC, and each of the clusters in the three-, four-, and five-cluster models had a mean PP for patients belonging to that cluster above 0.83. In the three-cluster model

For peer review only

the smallest cluster size was 430 (25%), in the four-cluster model it was 184 (11%) and the five-cluster model had a smallest cluster size of 142 (8%). Based on the cluster sizes, goodness-of-fit statistics, and interpretability, the four-cluster model was chosen as the optimal model.

Table 3 shows the probability of recorded receipt of each of the seven quality indicators for patients allocated to each cluster. Patients in cluster 1 (*n*=659, 38%) had a high probability of having pain and function assessment recorded (probabilities over 0.97) and of being given OA information and exercise advice (probabilities over 0.93). Patients' care within this cluster was recorded as having achieved a median of five indicators and considered for, but not achieved, a median of one further indicator. Cluster 1 was therefore labelled as having a *High* level of recorded quality of care. Cluster 2 (*n*=184, 11%; *Moderate*) had a high probability of pain and function assessment (probabilities over 0.95) and of consideration for (but not receipt of) physiotherapy and topical NSAID or paracetamol. They also had a high probability of being given or considered for OA information and exercise advice. Their recorded care achieved a median of three indicators and they were considered for care relating to a median of three further indicators. Cluster 3 (*n*=286, 17%; *Low*) had a high probability of pain and function assessment (probabilities over 0.87), and were likely to be prescribed or considered for paracetamol or topical NSAIDs but generally were not recorded as receiving or being considered for other indicators (received a median of three processes and considered for a median of one further). Cluster 4 (*n*=595, 35%; *None*) had low probabilities of a record of receiving or being considered for any indicator (received and considered median zero indicators).

Supplementary Table 1 compares the number of people in each cluster who were expected, based on the model, to receive each care process (identified by the indicators) and the number actually

For peer review only

recorded as receiving them. Differences between observed and expected values were small and generally related to distinguishing between care received compared to care considered.

Patient and clinician characteristics for each cluster are shown in Table 4 with results from the multinomial model comparing clusters in Table 5. Compared to the *None* cluster, patients in the *High* and *Moderate* clusters tended to consult with a clinician with a higher OA workload, consult multiple times, and have less total morbidity (Table 5). The patients with *High* level of recorded care were more likely to have diagnosed OA (adjusted RRR 1.81, 95% CI 1.41, 2.32) and less likely to have hand or foot clinical OA than patients in the *None* cluster, whilst patients in the *Moderate* cluster were less likely to have diagnosed OA (RRR 0.55, 95% CI 0.35, 0.85) or be overweight (RRR 0.57, 95% CI 0.39, 0.85), but more likely to have clinical OA in multiple sites (RRR 1.89, 95% CI 0.99, 3.59) than patients in the *None* cluster. Patients in the *Low* cluster were less likely than patients in the *None* cluster to have a single consultation (RRR 0.45, 95% CI 0.34, 0.60), have clinical OA in the foot (RRR 0.25, 95% CI 0.13, 0.51), or have multimorbidity.

Those in the *High* cluster had slightly higher levels of opioid prescription (36%; chi-squared test, p=0.06), oral NSAID prescription (20%; p=0.01), and recorded X-rays (22%; p<0.01) than patients in the other clusters, although differences between the *High* and *Low* clusters, in particular, were small (Table 6).

In those with a record of a pain assessment, patients in the *High* cluster were more likely to have recorded moderate or severe pain (70% vs 57% in the *Moderate* cluster and 64% in the *Low* cluster). The same pattern was seen for functional limitation although differences between clusters were smaller (Table 6).

For peer review only

Discussion

This study has identified four patterns of recorded primary care management of OA based on previously identified quality indicators of care. Just over a third of patients consulting for clinical OA had recorded care meeting the majority of quality indicators. Another third were not recorded as having received or been considered for any of these quality indicators. Factors associated with higher recorded quality of care included receiving an OA diagnosis, OA in the knee or hip rather than foot or hand, lower total morbidity burden, multiple consultations for clinical OA, and initial consultation with a clinician who was recorded as seeing more than the median number of OA patients. Previous evidence has demonstrated that guidelines for treatment of OA within primary care are not consistently adhered to [14-16]. The way in which receipt of different recommended care processes for OA are grouped within patients has not previously been investigated. In our study, 38% of the patients were recorded as having received a relatively large number of quality indicators and could be regarded as a group achieving the closest to optimal care based on these indicators (the *High* group). Care for members of two clusters (*Moderate* and *Low)* achieved some quality indicators overall but can be distinguished by the fact that information, advice (exercise, weight loss) and physiotherapy were more likely to be considered in the *Moderate* cluster than the *Low*. A third of patients were in the *None* cluster which demonstrated the weakest recorded quality of care with the majority of this group lacking recorded achievement or consideration of any indicator. The patients in the cluster with the best recorded care (*High*) were also more likely to receive other elements of care such as oral NSAIDs and referral for X-ray. NICE does not recommend routine use of X-ray for OA diagnosis and suggests that opioids and oral NSAIDS should be used only if topical NSAIDs and paracetamol do not relieve pain [3]. The greater use of these approaches in the *High* cluster may reflect worse severity of OA and this cluster did have slightly higher levels of clinician-recorded pain and functional limitation than those in the *Moderate* and

Low clusters. While one hypothesis may be that patients in the *High* cluster are given all possible care elements, this is unlikely to be the case as differences between clusters on the non-quality indicator elements of care were generally small, and most patients in the *High* cluster were not in receipt of these non-recommended approaches.

It is possible that the clinicians treating those in the *High* cluster were more engaged with, or more confident in managing OA. Confidence in OA management could be associated with confidence in OA diagnosis, which may explain the increased use of OA Read codes in these patients. Conversely, where OA Read codes were not given there may have been uncertainty about both diagnosis and management. Previous qualitative observational research of primary care consultations has identified confusion about the construct of OA, with family doctors tending not to use the term 'osteoarthritis' with patients but instead, normalising symptoms [17]. A formal diagnosis of OA, delivered explicitly, may be needed for holistic components of care such as patient education and self-management support to be offered [5,17]. Patients with greater morbidity received a lower recorded quality of care and this may be because they were (perhaps erroneously) considered less suitable for non-pharmacological and relatively safe pharmacological options. It is also possible that OA was given lower priority compared to their other problems [17,18]. Patients with foot (and to some extent hand) OA may also have been particularly susceptible to lower levels of recorded quality of care and this site has been less well-investigated with regard to effective interventions [19,20].

This is the first study known to the authors which examines patterns of quality of care of chronic conditions such as OA. Other analyses of recorded quality of care for OA have reported some influences on individual process measures. Broadbent et al. identified older age as being associated with reduced information provision but increased initial use of paracetamol and, where an oral

For peer review only

NSAID was prescribed, greater first-use of ibuprofen or a COX-2 selective NSAID; female sex was associated with increased information provision; severe OA was associated with increased pain and function assessment in the previous year [21]. Unlike in this analysis, Min et al. identified an association between multimorbidity (using a count of conditions) and better quality of care amongst vulnerable elders, some of whom had OA [22].

This study has important strengths. The study population was large and the practices were diverse with respect to urbanisation, staffing, deprivation, and size of registered population, implying good generalisability. Prescription recording is likely to be near-complete since most prescribing is electronic and use of the e-template mitigates against missing data from patients using over-the-counter pharmacological approaches. The e-template also facilitates enhanced data collection in general practice without incurring biases such as social desirability. LCA uses probabilistic modelling and finite mixture distributions to collect participants into clusters, which is a different method to traditional clustering techniques (e.g. cluster analysis). Given this, LCA should produce a lower misclassification rate and better statistical criteria for investigating model fit [23]. Whilst there was variation in quality of care between clinicians and practices [5], clustering effects of patients within clinicians was adjusted for through the multilevel model. There are some limitations in this analysis. Due to the inherent nature of EHR studies, the data extracted is a function of both the individual clinician's clinical and recording behaviours. It is therefore possible that some patients were misclassified as the lack of a record of a care process does not conclusively demonstrate that it did not occur. Compared to prescription recording, it is less certain how well-recorded referrals are. However, despite the limitations of EHR data, the differences in levels of prescribed analgesia between the clusters suggests there were real differences in care between the four clusters identified. Conversely, patients may have been coded as receiving some elements of care without

For peer review only

this necessarily having been conducted in a comprehensive or meaningful way. Triangulation of medical record indicators with patient-reported indicators would be needed to evaluate this further. Our assumption that those without a weight recorded were considered for weight loss advice was based on the increased likelihood of a weight recording if a patients appears overweight [10] but will have over-estimated the proportion of patients considered for weight loss advice. However, over 80% of patients did have a weight record. The association between multiple consultations for OA and clusters with higher recorded quality of care may reflect greater opportunity to provide and record care but may also have reflected a greater disease severity and healthcare need. Although we considered comorbidities, previous research has identified that OA may be discussed in complex consultations about multiple problems [17] and the length of time discussing OA in a consultation would likely be an important influence on the level of recorded care. It is also possible that those with recorded peripheral joint pain rather than recorded OA may not have OA, particularly in the foot [24]. The e-template itself was previously found to be associated with increased prescription of paracetamol and topical NSAIDs and so the patterns of care recorded may not be generalisable to practices not using the e-template [5].

Promotion of core interventions (information, exercise, and weight loss advice), alongside appropriate use of the relatively safe pharmacological options, remains an important strategy in the primary care management of OA but many patients receive few or none of these. This is particularly true for patients with higher levels of morbidity, or hand or foot OA. Whilst there is substantial variation in recorded care of OA, high quality care appears feasible given we found that over a third of patients with OA were recorded as receiving most core recommendations. A structured annual review for people with OA [25] as recommended by NICE [9] may help, possibly nurse-led, integrated, where appropriate, into a multimorbidity review. However, barriers to providing and

For peer review only

recording high quality care still need to be identified and mechanisms need to be explored to ensure appropriate delivery of care to all patients.

Acknowledgements

The authors would like to thank the OA Research Users' Group, NIHR West Midlands CRN Primary Care, and the network, health informatics, study coordinator, research nurse, and administrative staff at Keele University's Arthritis Research UK Primary Care Centre and Keele Clinical Trials Unit for all their support and assistance with this study. The authors would like to give special thanks to all of the staff and patients at the participating general practices, and the GP facilitators who provided support to the general practices involved in the study.

Author statement

HJ and LB performed the analysis and drafted and revised the paper. KPJ and JJE developed the analysis plan, cleaned the data, and drafted and revised the paper; KSD is PI for the study, led the design of the MOSAICS study, and revised the paper; EC, ZP and AF were involved in the interpretation of the findings and revised the paper. All authors have approved the final version.

Funding

This paper presents independent research funded by the National Institute for Health Research (NIHR) Programme Grant (RP-PG-0407-10386). The views expressed in this paper are those of the authors and not necessarily those of the NHS, the NIHR or the Department of Health. This research was also funded by the Arthritis Research UK Primary Care Centre grant (Grant Number 18139). HJ and LB were funded by a NIHR Research Methods Training Fellowship. KSD is part-funded by the NIHR Collaborations for Leadership in Applied Health Research and Care West Midlands and a

For peer review only

Knowledge Mobilisation Research Fellowship (KMRF-2014-03-002) from the NIHR. EC and JJE are Academic Clinical Lecturers in Primary Care funded by the NIHR; JJE was previously supported by an In-Practice Fellowship from the NIHR.

Competing interests

Prof. Jordan reports grants from National Institute for Health Research, grants from Arthritis Research UK, during the conduct of the study. Prof. Dziedic reports grants from Arthritis Research UK Centre in Primary Care grant, grants from National Institute for Health Research (NIHR) Porgramme Grant (RP-PG-0407-10386), during the conduct of the study; grants from Knowledge Mobilisation Research Fellowship (KMRF-2014-03-002), non-financial support from National Institute of Health and Care Excellence, other from Bone and Joint Decade 2015 Conference Oslo, non-financial support from National Institute of Health and Care Excellence Quality Standards, grants from EIT-Health, other from Osteoarthritis Research Society International, outside the submitted work; and Member of the NICE Osteoarthritis Guidelines Development Group CG 59 (2008) and CG 177 (2014). The other authors declare no competing interests.

Data access statement

The Centre has established data sharing arrangements to support joint publications and other research collaborations. Applications for access to anonymised data from our research databases are reviewed by the Centre's Data Custodian and Academic Proposal (DCAP) Committee and a decision regarding access to the data is made subject to the NRES ethical approval first provided for the study and to new analysis being proposed. Further information on our data sharing procedures can be found on the Centre's website

For peer review only

(http://www.keele.ac.uk/pchs/publications/datasharingresources/) or by emailing the Centre's data manager (primarycare.datasharing@keele.ac.uk).

For peer review only

References

1. Jordan KP, Joud A, Bergknut C, et al. International comparisons of the consultation prevalence of musculoskeletal conditions using population-based healthcare data from England and Sweden. Ann Rheum Dis 2014;**73**(1):212-8.
2. Vos T, Flaxman AD, Naghavi M, et al. Years lived with disability (YLDs) for 1160 sequelae of 289 diseases and injuries 1990-2010: a systematic analysis for the Global Burden of Disease Study 2010. Lancet 2012;**380**(9859):2163-96.
3. National Institute for Health & Care Excellence. NICE clinical guideline [CG177] Osteoarthritis: Care and management in adults. London: National Institute for Health & Care Excellence, 2014.
4. Hagen KB, Smedslund G, Osteras N, et al. Quality of Community-Based Osteoarthritis Care: A Systematic Review and Meta-Analysis. Arthritis Care Res (Hoboken) 2016;**68**(10):1443-52.
5. Edwards JJ, Jordan KP, Peat G, et al. Quality of care for OA: the effect of a point-of-care consultation recording template. Rheumatology (Oxford, England) 2015;**54**(5):844-53.
6. Dziedzic KS, Healey EL, Porcheret M, et al. Implementing the NICE osteoarthritis guidelines: a mixed methods study and cluster randomised trial of a model osteoarthritis consultation in primary care--the Management of OsteoArthritis In Consultations (MOSAICS) study protocol. Implement Sci 2014;**9**(1):95.
7. Edwards JJ, Khanna M, Jordan KP, et al. Quality indicators for the primary care of osteoarthritis: a systematic review. Ann Rheum Dis 2015;**74**(3):490-8.
8. National Institute for Health & Clinical Excellence. NICE clinical guideline [CG59] Osteoarthritis: the care and management of osteoarthritis in adults. London: National Institute for Health & Clinical Excellence, 2008.
9. National Institute for Health & Care Excellence. Quality standard for osteoarthritis (NICE quality standard 87). Secondary Quality standard for osteoarthritis (NICE quality standard 87) 2015. https://www.nice.org.uk/guidance/qs87.
10. Waring ME, Roberts MB, Parker DR, et al. Documentation and management of overweight and obesity in primary care. Journal of the American Board of Family Medicine : JABFM 2009;**22**(5):544-52.
11. Brilleman SL, Salisbury C. Comparing measures of multimorbidity to predict outcomes in primary care: a cross sectional study. Fam Pract 2013;**30**(2):172-8.
12. Magidson J, Vermunt JK. *Latent class models*. Thousand Oaks: Sage Publications, 2004.
13. Clark DB, Jones BL, Wood DS, et al. Substance use disorder trajectory classes: diachronic integration of onset age, severity, and course. Addictive behaviors 2006;**31**(6):995-1009.
14. Conrozier T, Marre JP, Payen-Champenois C, et al. National survey on the non-pharmacological modalities prescribed by French general practitioners in the treatment of lower limb (knee and hip) osteoarthritis. Adherence to the EULAR recommendations and factors influencing adherence. Clin Exp Rheumatol 2008;**26**(5):793-8.
15. DeHaan MN, Guzman J, Bayley MT, et al. Knee osteoarthritis clinical practice guidelines -- how are we doing? J Rheumatol 2007;**34**(10):2099.
16. Denoeud L, Mazieres B, Payen-Champenois C, et al. First line treatment of knee osteoarthritis in outpatients in France: adherence to the EULAR 2000 recommendations and factors influencing adherence. Ann Rheum Dis 2005;**64**(1):70-4.

For peer review only

17. Paskins Z, Sanders T, Croft PR, et al. The Identity Crisis of Osteoarthritis in General Practice: A Qualitative Study Using Video-Stimulated Recall. Ann Fam Med 2015;**13**(6):537-44.
18. Coxon D, Frisher M, Jinks C, et al. The relative importance of perceived doctor's attitude on the decision to consult for symptomatic osteoarthritis: a choice-based conjoint analysis study. BMJ Open 2015;**5**(10):e009625.
19. Iagnocco A, Rizzo C, Gattamelata A, et al. Osteoarthritis of the foot: a review of the current state of knowledge. Med Ultrason 2013;**15**(1):35-40.
20. Conaghan PG, Kloppenburg M, Schett G, et al. Osteoarthritis research priorities: a report from a EULAR ad hoc expert committee. Ann Rheum Dis 2014;**73**(8):1442-5.
21. Broadbent J, Maisey S, Holland R, et al. Recorded quality of primary care for osteoarthritis: an observational study. Br J Gen Pract 2008;**58**(557):839-43.
22. Min LC, Wenger NS, Fung C, et al. Multimorbidity is associated with better quality of care among vulnerable elders. Med Care 2007;**45**(6):480-8.
23. Hougaard P. Frailty models for survival data. Lifetime Data Anal 1995;**1**(3):255-73.
24. Thomas MJ, Roddy E, Rathod T, et al. Clinical diagnosis of symptomatic midfoot osteoarthritis: cross-sectional findings from the Clinical Assessment Study of the Foot. Osteoarthritis and cartilage / OARS, Osteoarthritis Research Society 2015;**23**(12):2094-101.
25. Healey EL, Main CJ, Ryan S, et al. A nurse-led clinic for patients consulting with osteoarthritis in general practice: development and impact of training in a cluster randomised controlled trial. BMC Fam Pract 2016;**17**(1):173.

For peer review only

Table 1: Quality Indicators and categories used for latent class analysis

Quality Indicator	Categories	Definition
Pain Assessed	Assessed	Recorded level of pain[a]
	Not Assessed	No entry recorded[a]
Function Assessed	Assessed	Recorded level of function[a]
	Not Assessed	No entry recorded[a]
OA Information	Given	Recorded written or verbal[a]
	Considered, but Not Given	Recorded not appropriate[a]
	Not Considered	No entry recorded[a]
Exercise Advice	Given	Recorded written or verbal[a]
	Considered, but Not Given	Recorded not appropriate[a]
	Not Considered	No entry recorded[a]
Weight loss Advice[c]	Given	Recorded written or verbal[a]
	Considered, but Not Given	Recorded not appropriate[a]
	Not Considered	No entry recorded[a]
Paracetamol or Topical NSAID	Prescribed	Either drug prescribed[b]
	Considered, but Not Prescribed	Neither drug prescribed but recorded tried, offered, patient declined, or not appropriate[a]
	Not Considered	Neither drug prescribed, recorded unknown or no entry recorded for both drugs[a]
Physiotherapy	Referred	Recorded referral[b]
	Considered, but Not Referred	No referral but recorded as offered, or not necessary or not appropriate[a]
	Not Considered	No referral, recorded not this time or no entry recorded[a]

[a] from e-template; [b] from routine records; [c] patients without a recorded BMI of ≥25 within the last 3 years were allocated to "Considered, but not given" category

For peer review only

Table 2: Latent class analysis goodness of fit statistics

Number of clusters	BIC	χ^2 goodness of fit	Population (%) of smallest cluster	Range of mean PP across clusters	*n* (%) with PP<0.7
1	20994.14	32978.08	1724 (100)	1.000	0 (0)
2	15160.57	3332.77	1071 (62)	0.992, 0.987	3 (<1)
3	14715.82	1727.74	430 (25)	0.906, 0.991	138 (8)
4	14627.48	1522.28	184 (11)	0.848, 0.994	157 (9)
5	14661.55	809.88	142 (8)	0.830, 0.993	207 (12)
6	14699.79	733.23	112 (6)	0.754, 0.996	257 (15)
7	14771.09	818.78	22 (1)	0.701, 0.996	267 (15)

BIC: Bayesian Information Criterion; PP: posterior probability

For peer review only

Table 3: Conditional item response probabilities for the quality indicators for each cluster

	Quality Indicators	Overall *n* (%)	Cluster *High* (*n*=659, 38%)	*Moderate* (*n*=184, 11%)	*Low* (*n*=286, 17%)	*None* (*n*=595, 35%)
Pain Assessment	Assessed	1092 (63)	0.978	0.961	0.922	0.014
	Not Assessed	632 (37)	0.022	0.039	0.078	0.987
Function Assessment	Assessed	1070 (62)	0.981	0.955	0.873	0.000
	Not Assessed	654 (38)	0.019	0.045	0.127	1.000
OA Information	Given	764 (44)	0.930	0.463	0.319	0.001
	Considered, Not Given	85 (5)	0.009	0.330	0.011	0.000
	Not Considered	875 (51)	0.062	0.207	0.670	1.000
Exercise Advice	Given	768 (45)	0.994	0.417	0.237	0.000
	Considered, Not Given	96 (6)	0.007	0.313	0.067	0.000
	Not Considered	860 (50)	0.000	0.270	0.696	1.000
Weight Advice	Given	536 (31)	0.593	0.115	0.089	0.000
	Considered, Not Given	153 (9)	0.298	0.733	0.347	0.441
	Not Considered	1035 (60)	0.109	0.152	0.564	0.559
Topical NSAID/ paracetamol	Prescribed	609 (35)	0.476	0.273	0.394	0.239
	Considered, Not Prescribed	570 (33)	0.496	0.641	0.406	0.004
	Not Considered	545 (32)	0.028	0.086	0.200	0.757
Physiotherapy	Referred	124 (7)	0.111	0.037	0.101	0.032
	Considered, Not Referred	532 (31)	0.559	0.732	0.080	0.000
	Not Considered	1068 (62)	0.330	0.230	0.819	0.968
Median count (IQR) Assessed/prescribed/given/referred			5 (4, 6)	3 (2, 3)	3 (2, 3)	0 (0, 1)
Median count (IQR) Considered			1 (1, 2)	3 (2, 4)	1 (0, 1)	0 (0, 1)

For peer review only

Table 4: Patient and clinician characteristics for each cluster

		Total *n* (%)	Cluster *High* (*n*=659)	*Moderate* (*n*=184)	*Low* (*n*=286)	*None* (*n*=595)
Age:	45-64	817	277 (34)	109 (13)	293 (43)	138 (17)
	65-74	442	213 (48)	20 (5)	144 (33)	65 (15)
	75-84	349	133 (38)	35 (10)	116 (33)	65 (19)
	85+	116	36 (31)	20 (17)	42 (36)	18 (6)
Gender:	Male	710	286 (40)	68 (10)	113 (16)	243 (34)
	Female	1014	373 (37)	116 (11)	173 (17)	352 (35)
BMI category:						
	Normal	315	111 (35)	54 (17)	48 (15)	102 (32)
	Overweight	1080	471 (44)	83 (8)	193 (18)	333 (31)
	Not recorded	329	77 (23)	47 (14)	45 (14)	160 (49)
Recorded joint pain only		1142	366 (32)	148 (13)	207 (18)	421 (37)
OA diagnosis		582	293 (50)	36 (6)	79 (14)	174 (30)
Site of OA:						
	Knee	855	359 (42)	80 (9)	149 (17)	267 (31)
	Hip	363	135 (37)	41 (11)	68 (19)	119 (33)
	Foot	125	30 (24)	15 (12)	10 (8)	70 (56)
	Hand	152	33 (22)	25 (16)	31 (20)	63 (41)
	Unspecified	99	30 (30)	8 (8)	16 (16)	45 (46)
	Multiple	130	72 (55)	15 (12)	12 (9)	31 (24)
Morbidity load[a]:						
BNF count	0-4	485	156 (32)	68 (14)	89 (18)	172 (36)
	5-9	578	240 (42)	56 (10)	99 (17)	183 (32)
	10+	661	263 (40)	60 (9)	98 (15)	240 (36)
Clinician OA workload[b]:						
	Below the median	197	41 (21)	16 (8)	36 (18)	104 (53)
	Above the median	1527	618 (41)	168 (11)	250 (16)	491 (32)
Median (IQR) no. of OA consultations[b]		1 (0, 1)	1 (1, 2)	1 (1, 2)	1 (1, 2)	1 (1, 1)
OA consultations[b]:						
	Multiple	532	250 (47)	63 (12)	99 (19)	120 (23)
	Single	1192	409 (34)	121 (10)	187 (16)	475 (40)
Consulter status:						
	Repeat	566	232 (41)	53 (9)	84 (15)	197 (35)
	New[c]	1158	427 (37)	131 (11)	202 (17)	398 (34)

[a] Number of BNF sections from which prescription was made in previous 12 months; [b] during six month period; [c] no clinical OA consultations within the previous 12 months

For peer review only

Table 5: Associations of patient and clinician characteristics with cluster membership

n= 1724		*High* vs *None* RRR[a] (95% CI)	*Moderate* vs *None* RRR[a] (95% CI)	*Low* vs *None* RRR[a] (95% CI)
Age:	45-64	1	1	1
	65-74	1.41 (1.07, 1.84)	0.45 (0.27, 0.74)	0.97 (0.69, 1.37)
	75-84	1.13 (0.83, 1.52)	1.02 (0.65, 1.60)	1.42 (0.99, 2.05)
	85+	0.91 (0.56, 1.47)	1.56 (0.85, 2.89)	1.24 (0.69, 2.23)
Gender:	Male	1	1	1
	Female	0.86 (0.69, 1.07)	1.03 (0.75, 1.43)	1.04 (0.80, 1.36)
BMI category:	Normal	1	1	1
	Overweight	1.20 (0.91, 1.60)	0.57 (0.39, 0.85)	1.33 (0.93, 1.90)
	Not recorded	0.39 (0.27, 0.56)	0.52 (0.33, 0.81)	0.52 (0.33, 0.82)
	Recorded joint pain only	1	1	1
	OA diagnosis	1.81 (1.41, 2.32)	0.55 (0.35, 0.85)	0.93 (0.68, 1.29)
Site of OA:	Knee	1	1	1
	Hip	0.86 (0.66, 1.14)	1.14 (0.76, 1.71)	1.04 (0.75, 1.44)
	Foot	0.38 (0.24, 0.60)	0.73 (0.39, 1.36)	0.25 (0.13, 0.51)
	Hand	0.45 (0.30, 0.70)	1.18 (0.70, 1.98)	0.88 (0.56, 1.39)
	Unspecified	0.48 (0.30, 0.80)	0.85 (0.38, 1.90)	0.74 (0.41, 1.34)
	Multiple	1.13 (0.75, 1.74)	1.89 (0.99, 3.59)	0.65 (0.34, 1.24)
Morbidity load[b]:				
BNF count	0-4	1	1	1
	5-9	0.95 (0.71, 1.26)	0.74 (0.50, 1.11)	0.75 (0.54, 1.06)
	10+	0.64 (0.47, 0.87)	0.55 (0.35, 0.86)	0.50 (0.35, 0.73)
Clinician OA workload[c]:				
	Below the median	1	1	1
	Above the median	2.90 (1.98, 4.25)	2.32 (1.33, 4.03)	1.46 (0.98, 2.18)
OA consultations[c]:				
	Multiple	1	1	1
	Single	0.43 (0.34, 0.54)	0.47 (0.33, 0.66)	0.45 (0.34, 0.60)
Consulter status:	Repeat	1	1	1
	New[d]	1.12 (0.89, 1.41)	1.09 (0.76, 1.55)	1.18 (0.88, 1.59)

[a] Relative risk ratio from multilevel multinomial regression (patients within initial clinician seen) adjusted for all presented covariates, *None* cluster is reference; [b] Number of BNF sections from which prescription was made in previous 12 months; [c] during six month period; [d] no clinical OA consultations within the previous 12 month

For peer review only

Table 6: Use of management processes other than those used as quality indicators, and recorded severity of pain and functional limitation, by cluster

n (column %)	Total *n* (%)	Cluster *High* (*n*=659)	*Moderate* (*n*=184)	*Low* (*n*=286)	None (*n*=595)	*p*-value[a]
Opioid Prescribed	557 (33)	236 (36)	54 (29)	94 (33)	173 (29)	0.06
Oral NSAID Prescribed	284 (17)	130 (20)	21 (11)	49 (17)	84 (14)	0.01
X-ray Requested	263 (15)	142 (22)	30 (16)	52 (18)	39 (7)	<0.01
n with pain record	1092	645	177	263	7	0.001[b]
No pain	16 (1)	4 (<1)	7 (4)	4 (2)	1	
Mild pain	348 (32)	187 (29)	69 (39)	91 (35)	1	
Moderate pain	582 (53)	357 (55)	84 (47)	136 (52)	5	
Severe pain	146 (13)	97 (15)	17 (10)	32 (12)	0	
n with function record	1070	646	174	250	0	0.004[b]
No limitation	101 (9)	46 (7)	29 (16)	26 (10)	0	
Mild limitation	456 (43)	276 (43)	73 (42)	107 (43)	0	
Moderate limitation	427 (40)	277 (43)	57 (33)	93 (37)	0	
Severe limitation	86 (8)	47 (7)	15 (9)	24 (10)	0	

[a]χ^2 test, [b]excluding *None* cluster

For peer review only

STROBE Statement—checklist of items that should be included in reports of observational studies

	Item No	Recommendation	Page
**Title and abstract**	1	(*a*) Indicate the study's design with a commonly used term in the title or the abstract	Title upload
		(*b*) Provide in the abstract an informative and balanced summary of what was done and what was found	Abstract upload
**Introduction**			
Background/rationale	2	Explain the scientific background and rationale for the investigation being reported	2
Objectives	3	State specific objectives, including any prespecified hypotheses	2
**Methods**			
Study design	4	Present key elements of study design early in the paper	2-3
Setting	5	Describe the setting, locations, and relevant dates, including periods of recruitment, exposure, follow-up, and data collection	3
Participants	6	(*a*) *Cohort study*—Give the eligibility criteria, and the sources and methods of selection of participants. Describe methods of follow-up<br>*Case-control study*—Give the eligibility criteria, and the sources and methods of case ascertainment and control selection. Give the rationale for the choice of cases and controls<br>*Cross-sectional study*—Give the eligibility criteria, and the sources and methods of selection of participants	3
		(*b*) *Cohort study*—For matched studies, give matching criteria and number of exposed and unexposed<br>*Case-control study*—For matched studies, give matching criteria and the number of controls per case	N/A
Variables	7	Clearly define all outcomes, exposures, predictors, potential confounders, and effect modifiers. Give diagnostic criteria, if applicable	3-5
Data sources/ measurement	8*	For each variable of interest, give sources of data and details of methods of assessment (measurement). Describe comparability of assessment methods if there is more than one group	3-5
Bias	9	Describe any efforts to address potential sources of bias	3
Study size	10	Explain how the study size was arrived at	N/A (sample size calculation was for the clinical outcomes reported elsewhere)
Quantitative variables	11	Explain how quantitative variables were handled in the analyses. If applicable, describe which groupings were chosen and why	4-5
Statistical methods	12	(*a*) Describe all statistical methods, including those used	5-6

For peer review only - http://bmjopen.bmj.com/site/about/guidelines.xhtml

to control for confounding	
(*b*) Describe any methods used to examine subgroups and interactions	N/A
(*c*) Explain how missing data were addressed	4
(*d*) *Cohort study*—If applicable, explain how loss to follow-up was addressed<br>*Case-control study*—If applicable, explain how matching of cases and controls was addressed<br>*Cross-sectional study*—If applicable, describe analytical methods taking account of sampling strategy	N/A
(*e*) Describe any sensitivity analyses	N/A

Continued on next page

For peer review only

For peer review only - http://bmjopen.bmj.com/site/about/guidelines.xhtml

Results			Page
Participants	13*	(a) Report numbers of individuals at each stage of study—eg numbers potentially eligible, examined for eligibility, confirmed eligible, included in the study, completing follow-up, and analysed	6
		(b) Give reasons for non-participation at each stage	N/A
		(c) Consider use of a flow diagram	N/A
Descriptive data	14*	(a) Give characteristics of study participants (eg demographic, clinical, social) and information on exposures and potential confounders	Table 4
		(b) Indicate number of participants with missing data for each variable of interest	N/A
		(c) *Cohort study*—Summarise follow-up time (eg, average and total amount)	N/A
Outcome data	15*	*Cohort study*—Report numbers of outcome events or summary measures over time	N/A
		Case-control study—Report numbers in each exposure category, or summary measures of exposure	N/A
		Cross-sectional study—Report numbers of outcome events or summary measures	Table 3
Main results	16	(*a*) Give unadjusted estimates and, if applicable, confounder-adjusted estimates and their precision (eg, 95% confidence interval). Make clear which confounders were adjusted for and why they were included	Table 5
		(*b*) Report category boundaries when continuous variables were categorized	Table 4, 5
		(*c*) If relevant, consider translating estimates of relative risk into absolute risk for a meaningful time period	N/A
Other analyses	17	Report other analyses done—eg analyses of subgroups and interactions, and sensitivity analyses	N/A
**Discussion**			
Key results	18	Summarise key results with reference to study objectives	8-9
Limitations	19	Discuss limitations of the study, taking into account sources of potential bias or imprecision. Discuss both direction and magnitude of any potential bias	11
Interpretation	20	Give a cautious overall interpretation of results considering objectives, limitations, multiplicity of analyses, results from similar studies, and other relevant evidence	12
Generalisability	21	Discuss the generalisability (external validity) of the study results	12
**Other information**			
Funding	22	Give the source of funding and the role of the funders for the present study and, if applicable, for the original study on which the present article is based	13

*Give information separately for cases and controls in case-control studies and, if applicable, for exposed and unexposed groups in cohort and cross-sectional studies.

Note: An Explanation and Elaboration article discusses each checklist item and gives methodological background and published examples of transparent reporting. The STROBE checklist is best used in conjunction with this article (freely available on the Web sites of PLoS Medicine at http://www.plosmedicine.org/, Annals of Internal Medicine at http://www.annals.org/, and Epidemiology at http://www.epidem.com/). Information on the STROBE Initiative is available at www.strobe-statement.org.

For peer review only

For peer review only - http://bmjopen.bmj.com/site/about/guidelines.xhtml

BMJ Open

Patterns of routine primary care for osteoarthritis in the UK: a cross-sectional electronic health records study

Journal:	*BMJ Open*
Manuscript ID	bmjopen-2017-019694.R1
Article Type:	Research
Date Submitted by the Author:	22-Nov-2017

Complete List of Authors:	Jackson, Holly; Keele University, Research Institute for Primary Care and Health Sciences; University of Nottingham, School of Mathematical Sciences<br>Barnett, Lauren; Keele University, Arthritis Research UK Primary Care Centre<br>Jordan, Kelvin; Keele University, Arthritis Research UK Primary Care Centre; Keele Clinical Trials Unit, David Weatherall Building<br>Dziedzic, Krysia; Arthritis Research Campaign National Primary Care Centre<br>Cottrell, Elizabeth ; Keele University, Research Institute for Primary Care & Health Sciences<br>Finney, Andrew; Keele University, Research Institute for Primary Care and Health Sciences<br>Paskins, Zoe; Keele University, Arthritis Research UK Primary Care Centre, Research Institute for Primary Care & Health Sciences<br>Edwards, John; Keele University Faculty of Health, Research Institute for Primary Care & Health Sciences

**Primary Subject Heading**:	General practice / Family practice
Secondary Subject Heading:	Rheumatology
Keywords:	Osteoarthritis, PRIMARY CARE, Quality in health care < HEALTH SERVICES ADMINISTRATION & MANAGEMENT, Latent class analysis

SCHOLARONE™
Manuscripts

Patterns of routine primary care for osteoarthritis: a cross-sectional electronic health records study

H Jackson[1,2], LA Barnett[1], KP Jordan[1,3], KS Dziedzic[1], E Cottrell[1], AG Finney[1], Z Paskins[1], JJ Edwards[1]

H Jackson: holly@gigglane.com

LA Barnett: l.a.barnett@keele.ac.uk

KP Jordan: k.p.jordan@keele.ac.uk

KS Dziedzic: k.s.dziedzic@keele.ac.uk

E Cottrell: e.cottrell@keele.ac.uk

AG Finney: a.finney@keele.ac.uk

Z Paskins: z.paskins@keele.ac.uk

JJ Edwards: j.j.edwards@keele.ac.uk

[1]Keele University Research Institute for Primary Care and Health Sciences; [2]University of Nottingham School of Mathematical Sciences; [3]Keele Clinical Trials Unit, David Weatherall Building, Keele University, Staffordshire, ST5 5BG

Correspondence to: Dr John Edwards, Arthritis Research UK Primary Care Centre, Research Institute for Primary Care & Health Sciences, Keele University, Staffordshire, ST5 5BG; e-mail: j.j.edwards@keele.ac.uk; tel. +44 (0) 1782 733977; fax.: +44 (0) 1782 734719

Word count: 3424

Running title: Patterns of primary care for osteoarthritis

For peer review only

Abstract

Objective

To determine common patterns of recorded primary care for osteoarthritis (OA), and patient and provider characteristics associated with the quality of recorded care.

Design

An observational study nested within a cluster-randomised controlled trial.

Setting

Eight UK general practices who were part of the Management of OSteoArthritis In ConsultationS (MOSAICS) study.

Participants

Patients recorded as consulting within the eight general practices for clinical OA.

Primary outcomes

Achievement of seven quality indicators of care (pain/function assessment, information provision, exercise/weight advice, analgesics, physiotherapy), recorded through an electronic template or routinely-recorded in the electronic healthcare records, were identified for patients aged ≥45 years consulting over a six-month period with clinical OA. Latent class analysis was used to cluster patients based on care received. Clusters were compared on patient and clinician-level characteristics.

Results

1724 patients (median by practice 183) consulted with clinical OA. Common patterns of recorded quality care were: Cluster 1 (38%, *High*) received most quality indicators of care; Cluster 2 (11%, *Moderate*) had pain and function assessment, and received or were considered for other indicators; Cluster 3 (17%, *Low*) had pain and function assessment, and received or were considered for paracetamol or topical NSAIDs; Cluster 4 (35%, *None*) had no recorded quality indicators. Patients

For peer review only

with higher levels of recorded care consulted a clinician who saw more OA patients, consulted multiple times, and had less morbidity. Those in the *High* cluster were more likely to have recorded diagnosed OA and have knee/hip OA.

Conclusions

Patterns of recorded care for OA fell into four natural clusters. Appropriate delivery of core interventions and relatively safe pharmacological options for OA are still not consistently recorded as provided in primary care. Further research to understand clinical recording behaviours and determine potential barriers to quality care alongside effective training for clinicians is needed.

Trial registration number: ISRCTN06984617

Keywords

Osteoarthritis, primary care, quality indicators, latent class analysis

Article summary

- This paper describes a novel use of latent class analysis to identify patterns of primary care for osteoarthritis (OA)
- The population studied was large and diverse, increasing generalisability, and based on a broad definition of clinical OA to reduce selection bias
- The analysis used some quality indicators of care newly-implemented in practices through an electronic template (pain/function assessment, information provision, exercise/weight advice, analgesics, physiotherapy), which may have increased the recorded quality of care compared to routine practice
- Four clusters of recorded care were identified: approximately one-third of patients had a high probability of delivery of most care processes whilst another third had a low probability of any such delivery. The remaining patients had a high probability of pain and function

For peer review only

assessment but were distinguished by the probability of delivery or consideration of other aspects of care.

Word count 3582

For peer review only

72 **Introduction**

73 Osteoarthritis (OA) is a common reason for adults aged ≥45 years to consult primary care. Annually, 74 in the UK, 4% of such adults are recorded as consulting in general practice for diagnosed OA, with 75 an additional 8% recorded with joint pain likely to be attributable to OA [1]. Osteoarthritis is a 76 common reason for disability, and was ranked the 11th biggest cause of disability by the 2010 77 Global Burden of Disease (GBD) [2].

78 The UK National Institute for Health and Care Excellence (NICE) OA management guidelines 79 recommend core strategies of information provision, physical activity and exercise, and weight 80 management, supplemented with use of relatively safe pharmacological management strategies 81 (for example, topical non-steroidal anti-inflammatory drugs [NSAIDs]), as necessary [3]. 82 Intensification of management should depend on response to these initial approaches. However, 83 there is evidence that patients diagnosed with OA do not receive care that is well aligned to 84 evidence-based recommendations and which may be overly dependent on pharmacological 85 methods [4].

86 We have previously identified variation between clinicians in recorded quality of individual 87 indicators of OA care [5]. However, patterns of OA care and factors linked with increased 88 probability of adherence to OA quality standards are less well-studied. Using electronic general 89 practice records data, the objectives of this study were to determine patterns of recorded primary 90 care for OA based on quality indicators, and to determine associations between higher-quality 91 recorded care and patient and clinician characteristics.

92 **Methods**

93 This analysis used data from the Management of OSteoArthritis In ConsultationS (MOSAICS) study 94 (Trial registration number ISRCTN06984617), approved by the North West Research Ethics

For peer review only

Committee, Cheshire (reference: 10/H1017/76) [6]. MOSAICS was a mixed-methods study, which investigated the effect of a model consultation for clinical OA. It was set within eight general practices in Cheshire, Shropshire and Staffordshire, UK. Practice eligibility has been reported elsewhere [6]. The current analysis, reported in line with STROBE guidelines, used anonymised information from the electronic health records (EHR) of these practices for the six-month baseline period before randomisation of practices to intervention or control arms [6]. At the beginning of the baseline period, a computerised template ("e-template", described below) was installed within the EHR and all practices continued with otherwise usual care until the end of the baseline period.

The study population was all patients aged ≥45 years registered with the eight general practices who consulted with clinical OA in the baseline six-month period. UK general practice utilises a system of Read codes (similar in principle to the International Classification of Diseases codes) to record symptoms, morbidities, and care processes [7]; within MOSAICS, clinical OA was defined as either a recorded OA Read code or a peripheral joint pain Read code for the hand, hip, knee, or foot, to reduce the potential for selection bias in clinician coding. Patients were allocated to an index clinician, being the clinician recording the first formally diagnosed (i.e. OA Read-coded) OA consultation in the baseline period or, if none, the first peripheral joint pain coded consultation in the same period.

For peer review only

Outcome measures were the seven indicators of quality of care for OA in general practice recorded in the EHR (Table 1). These could be entered into the EHR as routinely-recorded data or captured through the e-template. The identification and synthesis of appropriate quality indicators using a systematic review and NICE 2008 guidelines has previously been reported [5,8,9].

Achievement of prescribing and referral indicators (recorded prescription of topical NSAIDs or paracetamol, and onward physiotherapy referral) were determined from data in the routinely-

recorded component of the EHR and were determined to have been achieved if they were recorded within 14 days of any clinical OA consultation in the six-month period.

The e-template facilitated recording of achievement of indicators that are known to be poorly captured in routinely-recorded data [5]: (i) assessment of pain and function, (ii) provision or consideration of OA information, exercise advice, and weight loss advice, (iii) consideration of paracetamol or topical NSAID and (iv) consideration of physiotherapy referral. The entry of a code for clinical OA for a patient aged ≥45 years triggered the e-template. The design, interpretation, and effects of the e-template have previously been reported [5]. The clinicians could complete the e-template at any point throughout the consultation and could choose to complete all, some, or none of the e-template. The e-template has been endorsed by NICE to facilitate enhanced uptake of quality standards [10].

Data from the EHR (derived from both routinely-recorded data and the e-template) were amalgamated within the relevant quality indicator. For example, consideration of paracetamol and topical NSAIDs (entered using e-template) was combined with actual prescription of these agents (routinely-recorded data). Outcomes (Table 1) were dichotomous for pain and function assessments. For all other indicators, the possibilities were for the indicator to be *achieved*, *considered* (without record of having been delivered), or *not considered*. There is evidence that weight recording is more common in people who are overweight compared to those who are not [11]. To minimise the effect of missing data and to preserve the ability of the model to identify people who needed weight loss advice but were not recorded as receiving it, any patient recorded as being of normal weight or who did not have a weight recorded was allocated to *considered* for weight loss advice.

For peer review only

We investigated how patterns of care based on the quality indicators were associated with other OA care processes, recorded in the routine EHR within 14 days of any clinical OA consultation: prescriptions for oral NSAIDs and opioids, and relevant X-rays (hand, hip knee, or foot).

Factors potentially associated with patterns of quality of care that were considered were: patient age, gender, body mass index (BMI), the site of clinical OA, whether patients had multiple or a single consultation for clinical OA within the six-month time period, whether the patient was a new consulter (no clinical OA consultations within the previous 12 months) and total morbidity. Total morbidity was measured by a count of British National Formulary (BNF) subchapters from which prescriptions had been issued in the previous 12 months [12]. A proxy measure of OA workload for the patients' index clinician was determined by dichotomising the number of index clinical OA consultations at the median value (14) across clinicians.

Statistical analysis

Latent class analysis (LCA) was used to cluster patients into groups based on recorded achievement of the seven quality indicators. All patients within a cluster should have similar recorded care for their OA or joint pain, but care should differ between patients belonging to different clusters [13].

Latent class models were fitted, beginning with a one-cluster model where all the patients were assumed to have been given the same pattern of treatment of OA, up to a seven-cluster model. To determine the optimum number of clusters, we considered the Bayes Information Criterion [14] (BIC, whereby the lowest BIC indicated the best model) with the size of each cluster, and the interpretability of the model. Posterior probabilities (PP) for a patient (the probabilities of that patient belonging to each of the clusters within the model) were identified. The cluster that had the largest PP for a patient was the cluster that patient was assigned to. We used the mean PP for

For peer review only

162 patients allocated to each cluster to measure cluster separation; a mean PP of more than 0.7
163 indicated that the patients were clearly assigned to that specific cluster [15].

164 Using a two-level (patient within index clinician) multinomial multilevel logistic regression,
165 associations between the patient and clinician-level covariates and cluster membership were
166 estimated and reported as relative risk ratios (RRR) with 95% confidence intervals (CI). We also
167 used chi-squared tests to compare between clusters on levels of pain and functional limitation
168 (none, mild, moderate, severe) as recorded in the e-template.

169 Statistical analysis was performed using R studio version 3.3.0, and MLwiN version 2.35 for
170 Windows.

171 **Results**

172 During the six-month period, 1724 patients (median per practice *n*=183) consulted with a recorded
173 clinical OA code and triggered the e-template. All were included in the analysis. 1014 (59%) of these
174 were female, mean age was 66.1 years (SD: 11.9) and 582 (34%) patients were recorded with a
175 diagnosis of OA rather than peripheral joint pain. Among consulters, 50% were recorded as having
176 clinical OA at the knee, 21% at the hip, and the remainder with ankle/foot, wrist/hand, multisite, or
177 unspecified clinical OA.

178 As previously reported [5], pain (63%) and function (62%) assessment were the most commonly
179 achieved indicators. Recorded provision of OA information (44%), and exercise advice (45%) were
180 achieved in under half of patients, and weight loss advice in less than a third of patients (31%). 609
181 (35%) patients were prescribed paracetamol or topical NSAIDs. A referral for physiotherapy was
182 made in 7% of patients.

For peer review only

Table 2 shows the goodness-of-fit statistics for the LCA models with one to seven clusters. The four-cluster model gave the lowest BIC, and each of the clusters in the three-, four-, and five-cluster models had a mean PP for patients belonging to that cluster above 0.83. In the three-cluster model the smallest cluster size was 430 (25%), in the four-cluster model it was 184 (11%) and the five-cluster model had a smallest cluster size of 142 (8%). Based on the cluster sizes, goodness-of-fit statistics, and clinical interpretability, the four-cluster model was chosen as the optimal model.

Table 3 shows the probability of recorded receipt of each of the seven quality indicators for patients allocated to each cluster. Patients in cluster 1 (*n*=659, 38%) had a high probability of having pain and function assessment recorded (probabilities over 0.97) and of being given OA information and exercise advice (probabilities over 0.93). Patients' care within this cluster was recorded as having achieved a median of five indicators and considered for, but not achieved, a median of one further indicator. Cluster 1 was therefore labelled as having a *High* level of recorded quality of care. Cluster 2 (*n*=184, 11%; *Moderate*) had a high probability of pain and function assessment (probabilities over 0.95) and of consideration for (but not receipt of) physiotherapy and topical NSAID or paracetamol. They also had a high probability of being given or considered for OA information and exercise advice. Their recorded care achieved a median of three indicators and they were considered for care relating to a median of three further indicators. Cluster 3 (*n*=286, 17%; *Low*) had a high probability of pain and function assessment (probabilities over 0.87), and were likely to be prescribed or considered for paracetamol or topical NSAIDs but generally were not recorded as receiving or being considered for other indicators (received a median of three processes and considered for a median of one further). Cluster 4 (*n*=595, 35%; *None*) had low probabilities of a record of receiving or being considered for any indicator (received and considered median zero indicators).

For peer review only

Table 4 compares the number of people in each cluster who were expected, based on the model, to receive each care process (identified by the indicators) and the number actually recorded as receiving them. Differences between observed and expected values were small and generally related to distinguishing between care received compared to care considered. For example, in the pain assessment domain, there was no difference between the counts of observed and expected provision for the *High* and *Moderate* clusters, and a difference of only one patient in the *Low* and *None* clusters; for OA information provision, this was observed more frequently than expected for the *High* cluster (observed n=620 compared to 613 expected) but less frequently for the *Moderate* (59 vs. 85) and *Low* (85 vs. 91) clusters.

Patient and clinician characteristics for each cluster are shown in Table 5 with results from the multinomial model comparing clusters in Table 6. Compared to the *None* cluster, patients in the *High* and *Moderate* clusters tended to consult with a clinician with a higher OA workload, consult multiple times, and have less total morbidity (Table 6). The patients with *High* level of recorded care were more likely to have diagnosed OA (adjusted RRR 1.81, 95% CI 1.41, 2.32) and less likely to have hand or foot clinical OA than patients in the *None* cluster, whilst patients in the *Moderate* cluster were less likely to have diagnosed OA (RRR 0.55, 95% CI 0.35, 0.85) or be overweight (RRR 0.57, 95% CI 0.39, 0.85), but more likely to have clinical OA in multiple sites (RRR 1.89, 95% CI 0.99, 3.59) than patients in the *None* cluster. Patients in the *Low* cluster were less likely than patients in the *None* cluster to have a single consultation (RRR 0.45, 95% CI 0.34, 0.60), have clinical OA in the foot (RRR 0.25, 95% CI 0.13, 0.51), or have multimorbidity.

Those in the *High* cluster had slightly higher levels of opioid prescription (36%; chi-squared test, p=0.06), oral NSAID prescription (20%; p=0.01), and recorded X-rays (22%; p<0.01) than patients in

For peer review only

the other clusters, although differences between the *High* and *Low* clusters, in particular, were small (Table 7).

In those with a record of a pain assessment, patients in the *High* cluster were more likely to have recorded moderate or severe pain (70% vs 57% in the *Moderate* cluster and 64% in the *Low* cluster). The same pattern was seen for functional limitation although differences between clusters were smaller (Table 7).

Discussion

This study has identified four patterns of recorded primary care management of OA based on previously identified quality indicators of care. Just over a third of patients consulting for clinical OA had recorded care meeting the majority of quality indicators. Another third were not recorded as having received or been considered for any of these quality indicators. Factors associated with higher recorded quality of care included receiving an OA diagnosis, OA in the knee or hip rather than foot or hand, lower total morbidity burden, multiple consultations for clinical OA, and initial consultation with a clinician who was recorded as seeing more than the median number of OA patients. Previous evidence has demonstrated that guidelines for treatment of OA within primary care are not consistently adhered to [16-18]. The way in which receipt of different recommended care processes for OA are grouped within patients has not previously been investigated. In our study, 38% of the patients were recorded as having received a relatively large number of quality indicators and could be regarded as a group achieving the closest to optimal care based on these indicators (the *High* group). Care for members of two clusters (*Moderate* and *Low)* achieved some quality indicators overall but can be distinguished by the fact that information, advice (exercise, weight loss) and physiotherapy were more likely to be considered in the *Moderate* cluster than the *Low*. A third of patients were in the *None* cluster which demonstrated the weakest recorded quality

For peer review only

251 of care with the majority of this group lacking recorded achievement or consideration of any
252 indicator. The patients in the cluster with the best recorded care (*High*) were also more likely to
253 receive other elements of care such as oral NSAIDs and referral for X-ray. NICE does not
254 recommend routine use of X-ray for OA diagnosis and suggests that opioids and oral NSAIDS should
255 be used only if topical NSAIDs and paracetamol do not relieve pain [3]. The greater use of these
256 approaches in the *High* cluster may reflect worse severity of OA and this cluster did have slightly
257 higher levels of clinician-recorded pain and functional limitation than those in the *Moderate* and
258 *Low* clusters. While one hypothesis may be that patients in the *High* cluster are given all possible
259 care elements, this is unlikely to be the case as differences between clusters on the non-quality
260 indicator elements of care were generally small, and most patients in the *High* cluster were not in
261 receipt of these non-recommended approaches.

262 It is possible that the clinicians treating those in the *High* cluster were more engaged with, or more
263 confident in managing OA. Confidence in OA management could be associated with confidence in
264 OA diagnosis, which may explain the increased use of OA Read codes in these patients. Conversely,
265 where OA Read codes were not given there may have been uncertainty about both diagnosis and
266 management. Previous qualitative observational research of primary care consultations has
267 identified confusion about the construct of OA, with family doctors tending not to use the term
268 'osteoarthritis' with patients but instead, normalising symptoms [19]. A formal diagnosis of OA,
269 delivered explicitly, may be needed for holistic components of care such as patient education and
270 self-management support to be offered [5,19]. Patients with greater morbidity received a lower
271 recorded quality of care and this may be because they were (perhaps erroneously) considered less
272 suitable for non-pharmacological and relatively safe pharmacological options. It is also possible that
273 OA was given lower priority compared to their other problems [19,20]. Patients with foot (and to

For peer review only

some extent hand) OA may also have been particularly susceptible to lower levels of recorded quality of care and this site has been less well-investigated with regard to effective interventions [21,22].

This is the first study known to the authors which examines patterns of quality of care of chronic conditions such as OA. Other analyses of recorded quality of care for OA have reported some influences on individual process measures. Broadbent et al. identified older age as being associated with reduced information provision but increased initial use of paracetamol and, where an oral NSAID was prescribed, greater first-use of ibuprofen or a COX-2 selective NSAID; female sex was associated with increased information provision; severe OA was associated with increased pain and function assessment in the previous year [23]. Unlike in this analysis, Min et al. identified an association between multimorbidity (using a count of conditions) and better quality of care amongst vulnerable elders, some of whom had OA [24].

This study has important strengths. The study population was large and the practices were diverse with respect to urbanisation, staffing, deprivation, and size of registered population, implying good generalisability. Prescription recording is likely to be near-complete since most prescribing is electronic and use of the e-template mitigates against missing data from patients using over-the-counter pharmacological approaches. The e-template also facilitates enhanced data collection in general practice without incurring biases such as social desirability. LCA uses probabilistic modelling and finite mixture distributions to collect participants into clusters, which is a different method to traditional clustering techniques (e.g. cluster analysis). Given this, LCA should produce a lower misclassification rate and better statistical criteria for investigating model fit [25]. Whilst there was variation in quality of care between clinicians and practices [5], clustering effects of patients within clinicians was adjusted for through the multilevel model. There are some limitations in this analysis.

For peer review only

Due to the inherent nature of EHR studies, the data extracted is a function of both the individual clinician's clinical and recording behaviours. It is therefore possible that some patients were misclassified as the lack of a record of a care process does not conclusively demonstrate that it did not occur. Compared to prescription recording, it is less certain how well-recorded referrals are. However, despite the limitations of EHR data, the differences in levels of prescribed analgesia between the clusters suggests there were real differences in care between the four clusters identified. Conversely, patients may have been coded as receiving some elements of care without this necessarily having been conducted in a comprehensive or meaningful way. Triangulation of medical record indicators with patient-reported indicators would be needed to evaluate this further. Our assumption that those without a weight recorded were considered for weight loss advice was based on the increased likelihood of a weight recording if a patients appears overweight [11] but will have over-estimated the proportion of patients considered for weight loss advice. However, over 80% of patients did have a weight record. The association between multiple consultations for OA and clusters with higher recorded quality of care may reflect greater opportunity to provide and record care but may also have reflected a greater disease severity and healthcare need. Although we considered comorbidities, previous research has identified that OA may be discussed in complex consultations about multiple problems [19] and the length of time discussing OA in a consultation would likely be an important influence on the level of recorded care. It is also possible that those with recorded peripheral joint pain rather than recorded OA may not have OA, particularly in the foot [26]. The e-template itself was previously found to be associated with increased prescription of paracetamol and topical NSAIDs and so the patterns of care recorded may not be generalisable to practices not using the e-template [5].

For peer review only

Promotion of core interventions (information, exercise, and weight loss advice), alongside appropriate use of the relatively safe pharmacological options, remains an important strategy in the primary care management of OA but many patients receive few or none of these. This is particularly true for patients with higher levels of morbidity, or hand or foot OA. Whilst there is substantial variation in recorded care of OA, high quality care appears feasible given we found that over a third of patients with OA were recorded as receiving most core recommendations. A lack of a systematic approach to people with OA has previously been reported [27]. A structured annual review for people with OA [28] as recommended by NICE [10] may help. This may possibly be nurse-led and integrated, where appropriate, into a multimorbidity long-term condition review. However, causes of variation in providing and recording of high quality care still need to be identified and mechanisms need to be explored to ensure appropriate delivery of care to all patients.

Acknowledgements

The authors would like to thank the OA Research Users' Group, NIHR West Midlands CRN Primary Care, and the network, health informatics, study coordinator, research nurse, and administrative staff at Keele University's Arthritis Research UK Primary Care Centre and Keele Clinical Trials Unit for all their support and assistance with this study. The authors would like to give special thanks to all of the staff and patients at the participating general practices, and the GP facilitators who provided support to the general practices involved in the study.

Author statement

HJ and LB performed the analysis and drafted and revised the paper. KPJ and JJE developed the analysis plan, cleaned the data, and drafted and revised the paper; KSD is PI for the study, led the

For peer review only

design of the MOSAICS study, and revised the paper; EC, ZP and AF were involved in the interpretation of the findings and revised the paper. All authors have approved the final version.

Funding

This paper presents independent research funded by the National Institute for Health Research (NIHR) Programme Grant (RP-PG-0407-10386). The views expressed in this paper are those of the authors and not necessarily those of the NHS, the NIHR or the Department of Health. This research was also funded by the Arthritis Research UK Primary Care Centre grant (Grant Number 18139). HJ and LB were funded by a NIHR Research Methods Training Fellowship. KSD is part-funded by the NIHR Collaborations for Leadership in Applied Health Research and Care West Midlands and a Knowledge Mobilisation Research Fellowship (KMRF-2014-03-002) from the NIHR. EC and JJE are Academic Clinical Lecturers in Primary Care funded by the NIHR; JJE was previously supported by an In-Practice Fellowship from the NIHR.

Competing interests

Prof. Jordan reports grants from National Institute for Health Research, grants from Arthritis Research UK, during the conduct of the study. Prof. Dziedic reports grants from Arthritis Research UK Centre in Primary Care grant, grants from National Institute for Health Research (NIHR) Programme Grant (RP-PG-0407-10386), during the conduct of the study; grants from Knowledge Mobilisation Research Fellowship (KMRF-2014-03-002), non-financial support from National Institute of Health and Care Excellence, other from Bone and Joint Decade 2015 Conference Oslo, non-financial support from National Institute of Health and Care Excellence Quality Standards, grants from EIT-Health, other from Osteoarthritis Research Society International, outside the

For peer review only

submitted work; and Member of the NICE Osteoarthritis Guidelines Development Group CG 59 (2008) and CG 177 (2014). The other authors declare no competing interests.

Data access statement

The Centre has established data sharing arrangements to support joint publications and other research collaborations. Applications for access to anonymised data from our research databases are reviewed by the Centre's Data Custodian and Academic Proposal (DCAP) Committee and a decision regarding access to the data is made subject to the NRES ethical approval first provided for the study and to new analysis being proposed. Further information on our data sharing procedures can be found on the Centre’s website (http://www.keele.ac.uk/pchs/publications/datasharingresources/) or by emailing the Centre’s data manager (primarycare.datasharing@keele.ac.uk).

For peer review only

References

1. Jordan KP, Joud A, Bergknut C, et al. International comparisons of the consultation prevalence of musculoskeletal conditions using population-based healthcare data from England and Sweden. Ann Rheum Dis 2014;**73**(1):212-8.
2. Vos T, Flaxman AD, Naghavi M, et al. Years lived with disability (YLDs) for 1160 sequelae of 289 diseases and injuries 1990-2010: a systematic analysis for the Global Burden of Disease Study 2010. Lancet 2012;**380**(9859):2163-96.
3. National Institute for Health & Care Excellence. NICE clinical guideline [CG177] Osteoarthritis: Care and management in adults. London: National Institute for Health & Care Excellence, 2014.
4. Hagen KB, Smedslund G, Osteras N, et al. Quality of Community-Based Osteoarthritis Care: A Systematic Review and Meta-Analysis. Arthritis Care Res (Hoboken) 2016;**68**(10):1443-52.
5. Edwards JJ, Jordan KP, Peat G, et al. Quality of care for OA: the effect of a point-of-care consultation recording template. Rheumatology (Oxford, England) 2015;**54**(5):844-53.
6. Dziedzic KS, Healey EL, Porcheret M, et al. Implementing the NICE osteoarthritis guidelines: a mixed methods study and cluster randomised trial of a model osteoarthritis consultation in primary care--the Management of OsteoArthritis In Consultations (MOSAICS) study protocol. Implement Sci 2014;**9**(1):95.
7. Health and Social Care Information Centre. Read Codes. Secondary Read Codes 2017. https://digital.nhs.uk/article/1104/Read-Codes.
8. Edwards JJ, Khanna M, Jordan KP, et al. Quality indicators for the primary care of osteoarthritis: a systematic review. Ann Rheum Dis 2015;**74**(3):490-8.
9. National Institute for Health & Clinical Excellence. NICE clinical guideline [CG59] Osteoarthritis: the care and management of osteoarthritis in adults. London: National Institute for Health & Clinical Excellence, 2008.
10. National Institute for Health & Care Excellence. Quality standard for osteoarthritis (NICE quality standard 87). Secondary Quality standard for osteoarthritis (NICE quality standard 87) 2015. https://www.nice.org.uk/guidance/qs87.
11. Waring ME, Roberts MB, Parker DR, et al. Documentation and management of overweight and obesity in primary care. Journal of the American Board of Family Medicine : JABFM 2009;**22**(5):544-52.
12. Brilleman SL, Salisbury C. Comparing measures of multimorbidity to predict outcomes in primary care: a cross sectional study. Fam Pract 2013;**30**(2):172-8.
13. Magidson J, Vermunt JK. *Latent class models*. Thousand Oaks: Sage Publications, 2004.
14. Schwarz G. Estimating the Dimension of a Model. The Annals of Statistics 1978;**6**(2):461-4.
15. Clark DB, Jones BL, Wood DS, et al. Substance use disorder trajectory classes: diachronic integration of onset age, severity, and course. Addictive behaviors 2006;**31**(6):995-1009.
16. Conrozier T, Marre JP, Payen-Champenois C, et al. National survey on the non-pharmacological modalities prescribed by French general practitioners in the treatment of lower limb (knee and hip) osteoarthritis. Adherence to the EULAR recommendations and factors influencing adherence. Clin Exp Rheumatol 2008;**26**(5):793-8.
17. DeHaan MN, Guzman J, Bayley MT, et al. Knee osteoarthritis clinical practice guidelines -- how are we doing? J Rheumatol 2007;**34**(10):2099.

For peer review only

18. Denoeud L, Mazieres B, Payen-Champenois C, et al. First line treatment of knee osteoarthritis in outpatients in France: adherence to the EULAR 2000 recommendations and factors influencing adherence. Ann Rheum Dis 2005;**64**(1):70-4.
19. Paskins Z, Sanders T, Croft PR, et al. The Identity Crisis of Osteoarthritis in General Practice: A Qualitative Study Using Video-Stimulated Recall. Ann Fam Med 2015;**13**(6):537-44.
20. Coxon D, Frisher M, Jinks C, et al. The relative importance of perceived doctor's attitude on the decision to consult for symptomatic osteoarthritis: a choice-based conjoint analysis study. BMJ Open 2015;**5**(10):e009625.
21. Iagnocco A, Rizzo C, Gattamelata A, et al. Osteoarthritis of the foot: a review of the current state of knowledge. Med Ultrason 2013;**15**(1):35-40.
22. Conaghan PG, Kloppenburg M, Schett G, et al. Osteoarthritis research priorities: a report from a EULAR ad hoc expert committee. Ann Rheum Dis 2014;**73**(8):1442-5.
23. Broadbent J, Maisey S, Holland R, et al. Recorded quality of primary care for osteoarthritis: an observational study. Br J Gen Pract 2008;**58**(557):839-43.
24. Min LC, Wenger NS, Fung C, et al. Multimorbidity is associated with better quality of care among vulnerable elders. Med Care 2007;**45**(6):480-8.
25. Hougaard P. Frailty models for survival data. Lifetime Data Anal 1995;**1**(3):255-73.
26. Thomas MJ, Roddy E, Rathod T, et al. Clinical diagnosis of symptomatic midfoot osteoarthritis: cross-sectional findings from the Clinical Assessment Study of the Foot. Osteoarthritis and cartilage / OARS, Osteoarthritis Research Society 2015;**23**(12):2094-101.
27. Rosemann T, Wensing M, Joest K, et al. Problems and needs for improving primary care of osteoarthritis patients: the views of patients, general practitioners and practice nurses. BMC Musculoskelet Disord 2006;**7**:48.
28. Healey EL, Main CJ, Ryan S, et al. A nurse-led clinic for patients consulting with osteoarthritis in general practice: development and impact of training in a cluster randomised controlled trial. BMC Fam Pract 2016;**17**(1):173.

For peer review only

Table 1: Seven quality Indicators and categories used for latent class analysis

Quality Indicator	Categories	Definition
1. Pain assessed	Assessed	Recorded level of pain[a]
	Not assessed	No entry recorded[a]
2. Function assessed	Assessed	Recorded level of function[a]
	Not assessed	No entry recorded[a]
3. OA information	Given	Recorded written or verbal[a]
	Considered, but not given	Recorded not appropriate[a]
	Not considered	No entry recorded[a]
4. Exercise advice	Given	Recorded written or verbal[a]
	Considered, but not given	Recorded not appropriate[a]
	Not considered	No entry recorded[a]
5. Weight loss advice[c]	Given	Recorded written or verbal[a]
	Considered, but not given	Recorded not appropriate[a]
	Not considered	No entry recorded[a]
6. Paracetamol or topical NSAID	Prescribed	Either drug prescribed[b]
	Considered, but not prescribed	Neither drug prescribed but recorded tried, offered, patient declined, or not appropriate[a]
	Not considered	Neither drug prescribed, recorded unknown or no entry recorded for both drugs[a]
7. Physiotherapy	Referred	Recorded referral[b]
	Considered, but not referred	No referral but recorded as offered, or not necessary or not appropriate[a]
	Not considered	No referral, recorded not this time or no entry recorded[a]

[a] from e-template; [b] from routine records; [c] patients without a recorded BMI of ≥25 within the last 3 years were allocated to "Considered, but not given" category

For peer review only

Table 2: Latent class analysis goodness of fit statistics

Number of clusters	BIC	χ^2 goodness of fit	Population (%) of smallest cluster	Range of mean PP across clusters	*n* (%) with PP<0.7
1	20994.14	32978.08	1724 (100)	1.000	0 (0)
2	15160.57	3332.77	1071 (62)	0.992, 0.987	3 (<1)
3	14715.82	1727.74	430 (25)	0.906, 0.991	138 (8)
4	14627.48	1522.28	184 (11)	0.848, 0.994	157 (9)
5	14661.55	809.88	142 (8)	0.830, 0.993	207 (12)
6	14699.79	733.23	112 (6)	0.754, 0.996	257 (15)
7	14771.09	818.78	22 (1)	0.701, 0.996	267 (15)

BIC: Bayes Information Criterion; PP: posterior probability

For peer review only

Table 3: Conditional item response probabilities for the quality indicators for each cluster

	Quality Indicators	Overall<br>*n* (%)	Cluster<br>*High* (*n*=659, 38%)	 *Moderate* (*n*=184, 11%)	 *Low* (*n*=286, 17%)	 *None* (*n*=595, 35%)
Pain Assessment	Assessed	1092 (63)	0.978	0.961	0.922	0.014
	Not Assessed	632 (37)	0.022	0.039	0.078	0.987
Function Assessment	Assessed	1070 (62)	0.981	0.955	0.873	0.000
	Not Assessed	654 (38)	0.019	0.045	0.127	1.000
OA Information	Given	764 (44)	0.930	0.463	0.319	0.001
	Considered, Not Given	85 (5)	0.009	0.330	0.011	0.000
	Not Considered	875 (51)	0.062	0.207	0.670	1.000
Exercise Advice	Given	768 (45)	0.994	0.417	0.237	0.000
	Considered, Not Given	96 (6)	0.007	0.313	0.067	0.000
	Not Considered	860 (50)	0.000	0.270	0.696	1.000
Weight Advice	Given	536 (31)	0.593	0.115	0.089	0.000
	Considered, Not Given	153 (9)	0.298	0.733	0.347	0.441
	Not Considered	1035 (60)	0.109	0.152	0.564	0.559
Topical NSAID/ paracetamol	Prescribed	609 (35)	0.476	0.273	0.394	0.239
	Considered, Not Prescribed	570 (33)	0.496	0.641	0.406	0.004
	Not Considered	545 (32)	0.028	0.086	0.200	0.757
Physiotherapy	Referred	124 (7)	0.111	0.037	0.101	0.032
	Considered, Not Referred	532 (31)	0.559	0.732	0.080	0.000
	Not Considered	1068 (62)	0.330	0.230	0.819	0.968
Median count (IQR) Assessed/prescribed/given/referred			5 (4, 6)	3 (2, 3)	3 (2, 3)	0 (0, 1)
Median count (IQR) Considered			1 (1, 2)	3 (2, 4)	1 (0, 1)	0 (0, 1)

For peer review only

Table 4: Expected number compared to observed for each category of indicators, by cluster

		Cluster											
		**High** (*n*=659, 38%)			**Moderate** (*n*=184, 11%)			**Low** (*n*=286, 17%)			**None** (*n*=595, 35%)		
	**Quality Indicators**	**E**	**O**	**Δ**	**E**	**O**	**Δ**	**E**	**O**	**Δ**	**E**	**O**	**Δ**
Pain Assessment	Assessed (*n*=1092, 63%)	645	645	0	177	177	0	264	263	1	8	7	1
	*N*ot assessed (*n*=632, 37%)	14	14	0	7	7	0	22	23	-1	587	588	-1
Function Assessment	Assessed (*n*=1070, 62%)	646	646	0	176	174	2	250	250	0	0	0	0
	*N*ot assessed (*n*=655, 38 %)	13	13	0	8	10	-2	36	36	0	595	595	0
OA Information	Given (*n*=764, 44%)	613	620	-7	85	59	26	91	85	6	0	0	0
	Considered, *not* given (*n*=85, 5%)	6	3	3	61	81	-20	3	1	2	0	0	0
	*N*ot considered (*n*=875, 51%)	41	36	5	38	44	-6	192	200	-8	595	595	0
Exercise Advice	Given (*n*=768, 45%)	655	658	-3	77	53	24	68	57	11	0	0	0
	Considered, *not* given (*n*=96, 6%)	4	1	3	58	77	-19	19	18	1	0	0	0
	*N*ot considered (*n*=860, 50%)	0	0	0	50	54	-4	199	211	-12	595	595	0
Weight Advice	Given (*n*=536, 31%)	391	370	21	21	20	1	26	22	4	0	0	0
	Considered, *not* given (*n*= 153, 9%)	196	213	-17	135	140	-5	99	99	0	262	262	0
	*N*ot considered (*n*=1035, 60%)	72	76	-4	28	24	4	161	165	-4	333	333	0
Topical NSAID or paracetamol	Prescribed (*n*=609, 35%)	314	311	3	50	47	3	113	111	2	142	140	2
	Considered, *not* prescribed (*n*=570, 33%)	327	330	-3	118	119	-1	116	118	-2	2	3	-1
	*N*ot considered (*n*=545, 32%)	18	18	0	16	18	-2	57	57	0	450	452	-2
Physiotherapy	Referred (*n*=124, 7%)	73	69	4	7	6	1	29	30	-1	19	19	0
	Considered, *not* referred (*n*=532, 31%)	369	371	-2	135	147	-12	23	14	9	0	0	0
	*N*ot considered (*n*=1068, 62%)	218	219	-1	42	31	11	234	242	-8	576	576	0

E: expected number; **O**: observed number; **Δ**: difference

For peer review only

Table 5: Patient and clinician characteristics for each cluster

	Total *n* (%)	Cluster *High* (*n*=659)	*Moderate* (*n*=184)	*Low* (*n*=286)	*None* (*n*=595)
**Patient factors**					
**Age**					
45-64	817	277 (34)	109 (13)	293 (43)	138 (17)
65-74	442	213 (48)	20 (5)	144 (33)	65 (15)
75-84	349	133 (38)	35 (10)	116 (33)	65 (19)
85+	116	36 (31)	20 (17)	42 (36)	18 (6)
**Gender**					
Male	710	286 (40)	68 (10)	113 (16)	243 (34)
Female	1014	373 (37)	116 (11)	173 (17)	352 (35)
**BMI category:**					
Normal	315	111 (35)	54 (17)	48 (15)	102 (32)
Overweight	1080	471 (44)	83 (8)	193 (18)	333 (31)
Not recorded	329	77 (23)	47 (14)	45 (14)	160 (49)
**Diagnosis**					
Recorded with joint pain only	1142	366 (32)	148 (13)	207 (18)	421 (37)
OA diagnosis	582	293 (50)	36 (6)	79 (14)	174 (30)
**Site of OA:**					
Knee	855	359 (42)	80 (9)	149 (17)	267 (31)
Hip	363	135 (37)	41 (11)	68 (19)	119 (33)
Foot	125	30 (24)	15 (12)	10 (8)	70 (56)
Hand	152	33 (22)	25 (16)	31 (20)	63 (41)
Unspecified	99	30 (30)	8 (8)	16 (16)	45 (46)
Multiple	130	72 (55)	15 (12)	12 (9)	31 (24)
**Morbidity load[a]:**					
BNF count 0-4	485	156 (32)	68 (14)	89 (18)	172 (36)
5-9	578	240 (42)	56 (10)	99 (17)	183 (32)
10+	661	263 (40)	60 (9)	98 (15)	240 (36)
**Number of OA consultations[b]:**					
Multiple	532	250 (47)	63 (12)	99 (19)	120 (23)
Single	1192	409 (34)	121 (10)	187 (16)	475 (40)
Median (IQR) no. of OA consultations[b]	1 (0, 1)	1 (1, 2)	1 (1, 2)	1 (1, 2)	1 (1, 1)
**Consulter status:**					
Repeat	566	232 (41)	53 (9)	84 (15)	197 (35)
New[c]	1158	427 (37)	131 (11)	202 (17)	398 (34)
**Clinician factors**					
**Clinician OA workload[b]:**					
Below the median	197	41 (21)	16 (8)	36 (18)	104 (53)
Above the median	1527	618 (41)	168 (11)	250 (16)	491 (32)

[a] Number of BNF subchapters from which prescription was made in previous 12 months; [b] during six month period; [c] no clinical OA consultations within the previous 12 months

For peer review only

Table 6: Associations of patient and clinician characteristics with cluster membership

n= 1724	*High* vs *None* RRR[a] (95% CI)	*Moderate* vs *None* RRR[a] (95% CI)	*Low* vs *None* RRR[a] (95% CI)
**Patient factors**			
**Age**			
45-64	1	1	1
65-74	1.41 (1.07, 1.84)	0.45 (0.27, 0.74)	0.97 (0.69, 1.37)
75-84	1.13 (0.83, 1.52)	1.02 (0.65, 1.60)	1.42 (0.99, 2.05)
85+	0.91 (0.56, 1.47)	1.56 (0.85, 2.89)	1.24 (0.69, 2.23)
**Gender**			
Male	1	1	1
Female	0.86 (0.69, 1.07)	1.03 (0.75, 1.43)	1.04 (0.80, 1.36)
**BMI category**			
Normal	1	1	1
Overweight	1.20 (0.91, 1.60)	0.57 (0.39, 0.85)	1.33 (0.93, 1.90)
Not recorded	0.39 (0.27, 0.56)	0.52 (0.33, 0.81)	0.52 (0.33, 0.82)
**Diagnosis**			
Recorded with joint pain only	1	1	1
OA diagnosis	1.81 (1.41, 2.32)	0.55 (0.35, 0.85)	0.93 (0.68, 1.29)
**Site of OA**			
Knee	1	1	1
Hip	0.86 (0.66, 1.14)	1.14 (0.76, 1.71)	1.04 (0.75, 1.44)
Foot	0.38 (0.24, 0.60)	0.73 (0.39, 1.36)	0.25 (0.13, 0.51)
Hand	0.45 (0.30, 0.70)	1.18 (0.70, 1.98)	0.88 (0.56, 1.39)
Unspecified	0.48 (0.30, 0.80)	0.85 (0.38, 1.90)	0.74 (0.41, 1.34)
Multiple	1.13 (0.75, 1.74)	1.89 (0.99, 3.59)	0.65 (0.34, 1.24)
**Morbidity load[b]:**			
BNF count 0-4	1	1	1
5-9	0.95 (0.71, 1.26)	0.74 (0.50, 1.11)	0.75 (0.54, 1.06)
10+	0.64 (0.47, 0.87)	0.55 (0.35, 0.86)	0.50 (0.35, 0.73)
**Number of OA consultations[c]**			
Multiple	1	1	1
Single	0.43 (0.34, 0.54)	0.47 (0.33, 0.66)	0.45 (0.34, 0.60)
**Consulter status**			
Repeat	1	1	1
New[d]	1.12 (0.89, 1.41)	1.09 (0.76, 1.55)	1.18 (0.88, 1.59)
**Clinician factors**			
**Clinician OA workload[c]**			
Below the median	1	1	1
Above the median	2.90 (1.98, 4.25)	2.32 (1.33, 4.03)	1.46 (0.98, 2.18)

[a] Relative risk ratio from multilevel multinomial regression (patients within initial clinician seen) adjusted for all presented covariates, *None* cluster is reference; [b] Number of BNF subchapters from which prescription was made in previous 12 months; [c] during six month period; [d] no clinical OA consultations within the previous 12 month

For peer review only

Table 7: Use of management processes other than those used as quality indicators, and recorded severity of pain and functional limitation, by cluster

n (column %)	Total *n* (%)	Cluster *High* (*n*=659)	*Moderate* (*n*=184)	*Low* (*n*=286)	None (*n*=595)	*p*-value[a]
Opioid Prescribed	557 (33)	236 (36)	54 (29)	94 (33)	173 (29)	0.06
Oral NSAID Prescribed	284 (17)	130 (20)	21 (11)	49 (17)	84 (14)	0.01
X-ray Requested	263 (15)	142 (22)	30 (16)	52 (18)	39 (7)	<0.01
n with pain record	1092	645	177	263	7	0.001[b]
No pain	16 (1)	4 (<1)	7 (4)	4 (2)	1	
Mild pain	348 (32)	187 (29)	69 (39)	91 (35)	1	
Moderate pain	582 (53)	357 (55)	84 (47)	136 (52)	5	
Severe pain	146 (13)	97 (15)	17 (10)	32 (12)	0	
n with function record	1070	646	174	250	0	0.004[b]
No limitation	101 (9)	46 (7)	29 (16)	26 (10)	0	
Mild limitation	456 (43)	276 (43)	73 (42)	107 (43)	0	
Moderate limitation	427 (40)	277 (43)	57 (33)	93 (37)	0	
Severe limitation	86 (8)	47 (7)	15 (9)	24 (10)	0	

[a]χ^2 test, [b] excluding *None* cluster

For peer review only

STROBE Statement—checklist of items that should be included in reports of observational studies

	Item No	Recommendation	Page
**Title and abstract**	1	(*a*) Indicate the study's design with a commonly used term in the title or the abstract	Title upload
		(*b*) Provide in the abstract an informative and balanced summary of what was done and what was found	Abstract upload
**Introduction**			
Background/rationale	2	Explain the scientific background and rationale for the investigation being reported	2
Objectives	3	State specific objectives, including any prespecified hypotheses	2
**Methods**			
Study design	4	Present key elements of study design early in the paper	2-3
Setting	5	Describe the setting, locations, and relevant dates, including periods of recruitment, exposure, follow-up, and data collection	3
Participants	6	(*a*) *Cohort study*—Give the eligibility criteria, and the sources and methods of selection of participants. Describe methods of follow-up<br>*Case-control study*—Give the eligibility criteria, and the sources and methods of case ascertainment and control selection. Give the rationale for the choice of cases and controls<br>*Cross-sectional study*—Give the eligibility criteria, and the sources and methods of selection of participants	3
		(*b*) *Cohort study*—For matched studies, give matching criteria and number of exposed and unexposed<br>*Case-control study*—For matched studies, give matching criteria and the number of controls per case	N/A
Variables	7	Clearly define all outcomes, exposures, predictors, potential confounders, and effect modifiers. Give diagnostic criteria, if applicable	3-5
Data sources/ measurement	8*	For each variable of interest, give sources of data and details of methods of assessment (measurement). Describe comparability of assessment methods if there is more than one group	3-5
Bias	9	Describe any efforts to address potential sources of bias	3
Study size	10	Explain how the study size was arrived at	N/A (sample size calculation was for the clinical outcomes reported elsewhere)
Quantitative variables	11	Explain how quantitative variables were handled in the analyses. If applicable, describe which groupings were chosen and why	4-5
Statistical methods	12	(*a*) Describe all statistical methods, including those used	5-6

For peer review only - http://bmjopen.bmj.com/site/about/guidelines.xhtml

	to control for confounding	
	(*b*) Describe any methods used to examine subgroups and interactions	N/A
	(*c*) Explain how missing data were addressed	4
	(*d*) *Cohort study*—If applicable, explain how loss to follow-up was addressed<br>*Case-control study*—If applicable, explain how matching of cases and controls was addressed<br>*Cross-sectional study*—If applicable, describe analytical methods taking account of sampling strategy	N/A
	(*e*) Describe any sensitivity analyses	N/A

Continued on next page

For peer review only

Results			Page
Participants	13*	(a) Report numbers of individuals at each stage of study—eg numbers potentially eligible, examined for eligibility, confirmed eligible, included in the study, completing follow-up, and analysed	6
		(b) Give reasons for non-participation at each stage	N/A
		(c) Consider use of a flow diagram	N/A
Descriptive data	14*	(a) Give characteristics of study participants (eg demographic, clinical, social) and information on exposures and potential confounders	Table 4
		(b) Indicate number of participants with missing data for each variable of interest	N/A
		(c) *Cohort study*—Summarise follow-up time (eg, average and total amount)	N/A
Outcome data	15*	*Cohort study*—Report numbers of outcome events or summary measures over time	N/A
		Case-control study—Report numbers in each exposure category, or summary measures of exposure	N/A
		Cross-sectional study—Report numbers of outcome events or summary measures	Table 3
Main results	16	(*a*) Give unadjusted estimates and, if applicable, confounder-adjusted estimates and their precision (eg, 95% confidence interval). Make clear which confounders were adjusted for and why they were included	Table 5
		(*b*) Report category boundaries when continuous variables were categorized	Table 4, 5
		(*c*) If relevant, consider translating estimates of relative risk into absolute risk for a meaningful time period	N/A
Other analyses	17	Report other analyses done—eg analyses of subgroups and interactions, and sensitivity analyses	N/A
**Discussion**			
Key results	18	Summarise key results with reference to study objectives	8-9
Limitations	19	Discuss limitations of the study, taking into account sources of potential bias or imprecision. Discuss both direction and magnitude of any potential bias	11
Interpretation	20	Give a cautious overall interpretation of results considering objectives, limitations, multiplicity of analyses, results from similar studies, and other relevant evidence	12
Generalisability	21	Discuss the generalisability (external validity) of the study results	12
**Other information**			
Funding	22	Give the source of funding and the role of the funders for the present study and, if applicable, for the original study on which the present article is based	13

*Give information separately for cases and controls in case-control studies and, if applicable, for exposed and unexposed groups in cohort and cross-sectional studies.

Note: An Explanation and Elaboration article discusses each checklist item and gives methodological background and published examples of transparent reporting. The STROBE checklist is best used in conjunction with this article (freely available on the Web sites of PLoS Medicine at http://www.plosmedicine.org/, Annals of Internal Medicine at http://www.annals.org/, and Epidemiology at http://www.epidem.com/). Information on the STROBE Initiative is available at www.strobe-statement.org.

For peer review only
